# P-1157. In Vitro Antimicrobial Activity of Cefepime-Taniborbactam Against Molecularly Characterized Enterobacterales and P. aeruginosa Collected Worldwide from 2018-2023

**DOI:** 10.1093/ofid/ofaf695.1350

**Published:** 2026-01-11

**Authors:** Mark G Wise, Meredith Hackel, Daniel F Sahm

**Affiliations:** IHMA, Schaumburg, IL; IHMA, Schaumburg, IL; IHMA, Schaumburg, IL

## Abstract

**Background:**

The novel β-lactamase inhibitor, taniborbactam, is notable for its broad-spectrum inhibitory activity including the ability to inhibit serine-, and NDM- and VIM-type metallo-β-lactamases (MBLs). Taniborbactam potentiates cefepime against cephalosporin- and carbapenem-resistant (R) Enterobacterales (EB) and *Pseudomonas aeruginosa* (PA). The activities of cefepime-taniborbactam (FTB) and comparators were evaluated against a large global collection of clinical isolates of EB and PA with defined β-lactamase carriage.

**Figure:**
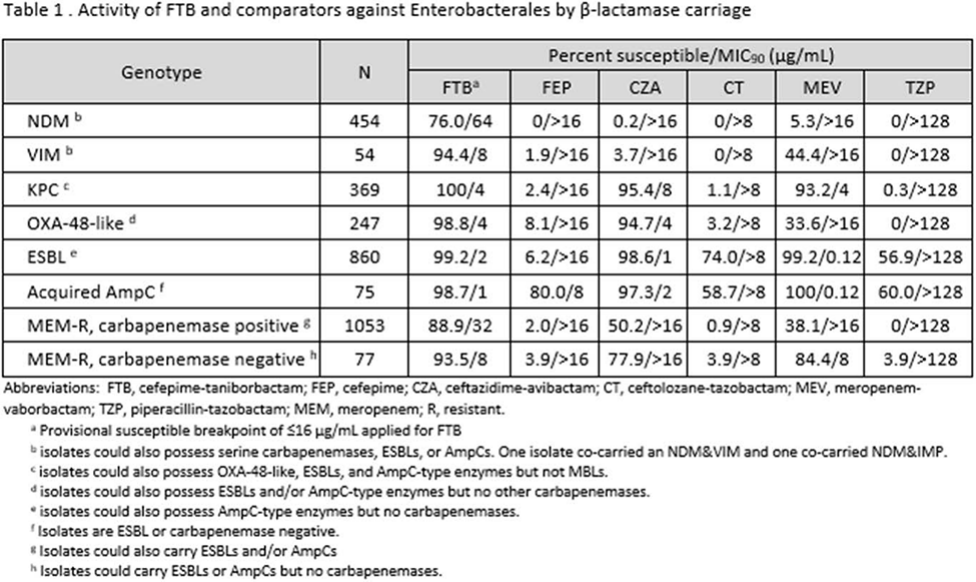

**Figure:**
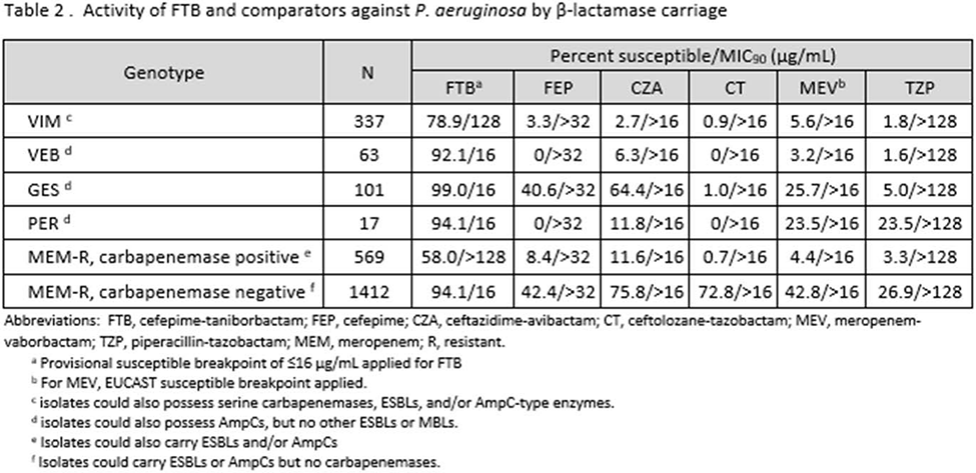

**Methods:**

MICs of FTB (taniborbactam fixed at 4 µg/mL) and comparators were determined using the CLSI reference method against EB (n=23,624) and PA (n=9,427) collected from 351 clinical laboratories in 62 countries from 2018-2023 and interpreted with CLSI 2025 breakpoints. For FTB, a provisional susceptible MIC breakpoint of ≤16 µg/mL was used for comparative purposes. Organisms with FTB MIC ≥16 µg/mL, those R to meropenem, and approximately 25% of EB susceptible to meropenem, but with ceftazidime or cefepime MIC ≥2 µg/mL, were screened for acquired β-lactamases by either PCR or WGS.

**Results:**

FTB was the only agent with activity against NDM-harboring EB (Table 1; 76.0% inhibited at ≤16 µg/mL). 94.4% of VIM-carrying EB were inhibited by FTB, 50 percentage points higher than meropenem-vaborbactam. FTB also exhibited potent activity versus KPC-, OXA-48-like-, ESBL- and AmpC-harboring isolates, with >98% of each group inhibited at ≤16 µg/mL. FTB at ≤16 µg/mL inhibited 88.9% of the carbapenemase-carrying meropenem-R EB and 93.5% of those without a carbapenemase. Against PA, FTB was the sole agent with activity versus isolates carrying VIM-type MBLs (78.9% inhibited at ≤16 µg/mL) and displayed high levels of activity against isolates carrying ESBLs, inhibiting >92% of the population of VEB-, GES- and PER- carriers (Table 2). FTB at ≤16 µg/ml inhibited 94.1% of meropenem-R PA isolates without a detected carbapenemase.

**Conclusion:**

Taniborbactam greatly enhanced cefepime *in vitro* activity against both EB and PA carrying serine- and metallo-β-lactamases. These findings support the continued development of FTB as a potential new therapeutic agent for use against β-lactamase-harboring Gram-negative pathogens.

**Disclosures:**

Mark G Wise, PhD, IHMA: Employee

